# Editorial: Case reports in general cardiovascular medicine: 2023

**DOI:** 10.3389/fcvm.2024.1461761

**Published:** 2024-08-22

**Authors:** Hongyun Wang, Qianwen Wu, Wei Chen, Leonardo Roever, Pietro Enea Lazzerini, Junjie Xiao

**Affiliations:** ^1^ Cardiac Regeneration and Ageing Lab, Institute of Cardiovascular Sciences, Shanghai Engineering Research Center of Organ Repair, Joint International Research Laboratory of Biomaterials and Biotechnology in Organ Repair (Ministry of Education), School of Life Science, Shanghai University, Shanghai, China; ^2^ Department of Rheumatology, The First Affiliated Hospital of Nanjing Medical University, Nanjing, China; ^3^ Emergency Department, Shanghai Tongji Hospital, Tongji University School of Medicine, Shanghai, China; ^4^ Department of Clinical Research, Brazilian Evidence-Based Health Network, Uberlândia, Brazil; ^5^ Gilbert and Rose-Marie Chagoury School of Medicine, Lebanese American University, Beirut, Lebanon; ^6^ Department of Medical Sciences, Surgery and Neurosciences, Division of Internal Medicine and Geriatrics, Electroimmunology Unit, University of Siena, Policlinico “Le Scotte”, Siena, Italy

**Keywords:** case reports, diagnosis, treatment, prognosis, cardiovascular medicine, editorial

**Editorial on the Research Topic**
Case reports in general cardiovascular medicine: 2023

Cardiovascular diseases (CVD), a big threat to public health worldwide, have been considered as a leading noninfectious killer to individuals. Understanding the mechanisms and developing effective treatments for CVD is a top priority (1–3).

In this editorial, we took insight into case reports featured in the Frontiers in Cardiovascular Medicine Research Topic “Case Reports in General Cardiovascular Medicine: 2023.” This special issue collects over 20 cases that cover diagnosis, management and outcomes of complex and rare CVD. This special issue has garnered widespread attention with more than twenty-five thousand views and including more than four thousand downloads. Table 1 presents the Case Reports published in the General Cardiovascular Medicine Section in the year 2023. By sharing these unique experiences, the authors contributed to enhancing medical knowledge and improving clinical care.

**Table 1:** Metrics (on July 8, 2024) of the articles published in case reports in general cardiovascular medicine: 2023.

Title	Authors	Views	Downloads
Case report: The presence of third-degree atrioventricular block caused by pulmonary embolism masquerading as acute ST-segment elevation myocardial infarction	Ma et al.	633	322
Successful cardiopulmonary resuscitation of cardiac arrest induced by massive pulmonary embolism under general anesthesia: a case report	Li and Cai	1,713	417
Tale of two hearts: a TNNT2 hypertrophic cardiomyopathy case report	Pham et al.	3,200	482
A case report of multiple artery pseudoaneurysms associated with SARS-CoV-2	Zhang et al.	1,200	359
Coronary artery mycotic aneurysm in a patient suffering from subacute endocarditis: a case report and literature review	Hali et al.	902	440
Cardiac pacemaker–related endocarditis complicated with pulmonary embolism: Case report	Šačić et al.	1,400	267
Case Report: Postmortem brain and heart pathology unveiling the pathogenesis of coexisting acute ischemic stroke and electrocardiographic abnormality	Hattori et al.	1,150	516
A novel compound heterozygous variant in ALPK3 induced hypertrophic cardiomyopathy: a case report	Li et al.	1,326	548
Case Report: Hypertrophic cardiomyopathy with recurrent episodes of ventricular fibrillation and concurrent sinus arrest	Hamidi et al.	1,195	358
Case Report: Early diagnosis and bevacizumab-based chemotherapy for primary pericardial mesothelioma: a case with occupational asbestos exposure history	Wang et al.	1,025	279
Case Report: Flurbiprofen-induced Type I Kounis syndrome	Tang et al.	811	286
Subacute hemorrhagic pericardial tamponade after COVID-19 infection mimicking carcinomatous pericarditis: a case report	Yamamoto et al.	1,900	240
A case report on pheochromocytoma mimicking as fulminant myocarditis—a diagnostic challenge	Cheng et al.	667	237
Case Report: Thrombus aspiration and *in situ* thrombolysis via a Guidezilla guide extension catheter in a patient with high-risk pulmonary embolism	Ding et al.	706	105
Case Report: Three cases of clinically suspected viral myocarditis with recovery of left ventricular dysfunction	Brown et al.	519	129
Venoarterial extracorporeal membrane oxygenation for vasoplegic shock after treprostinil refill of an implanted intravenous pump: a case report	Valencia et al.	1,200	435
A Case Report of Mycoplasma pneumoniae-induced fulminant myocarditis in a 15-year-old male leading to cardiogenic shock and electrical storm	Zhu et al.	746	133
Case Report: A case of Kounis syndrome induced by iodine contrast agent during coronary angiography	Sun et al.	722	261
Sudden death associated with delayed cardiac rupture: case report and literature review	Tinzin et al.	520	102
Old woman with Sheehan's syndrome suffered severe hyponatremia following percutaneous coronary intervention: a case report and review of literature	Gao et al.	657	179

Pulmonary embolism (PE) is a life-threatening condition resulting from a blockage in the pulmonary arteries with big challenges, which has been the focus of several compelling cases (4). Different with the typical symptoms such as chest pain, rapid breathing, hemoptysis, and syncope, Ma et al.'s exceptional case report described a rare occurrence of PE by which manifested with third-degree atrioventricular block and ST-segment elevation. The patient's history of cerebral hemorrhage led to a complexity of diagnosis, emphasizing the importance of a comprehensive assessment of all clinical findings to decrease misdiagnosis rates. Šačić et al. presented a case of woman with an implanted pacemaker by which led to infective endocarditis, affecting the right atrium and ventricle, further complicated by pulmonary embolism. This case highlighted the growing incidence of endocarditis associated with cardiac devices, particularly in patients with complex medical backgrounds requiring immunosuppressive therapy. An additional striking case shared by Li et al. depicted a sudden and severe PE episode that resulted in cardiac arrest in a 59-year-old male undergoing a routine femoral fracture reduction procedure under general anesthesia. Prompt diagnosis facilitated by computed tomography pulmonary angiography (CTPA) and subsequent thrombolytic therapy proved pivotal in successful resuscitation, underscoring the critical need for timely intervention in acute PE situations. Moreover, Ding et al. introduced an innovative approach utilizing catheter-directed thrombolysis and thrombus aspiration with the Guidezilla guide extension catheter to manage a massive PE case ineligible for systemic thrombolysis. This novel intervention strategy holds significant potential, particularly in resource-constrained settings. Collectively, these case studies illuminated the landscape of PE management, emphasizing the need for personalized care, advanced diagnostic techniques, and innovative therapeutic interventions to optimize patient outcomes.

The research topic on hypertrophic cardiomyopathy (HCM) illuminates the intricate and multifaceted nature of this heritable cardiac disorder, which is predominantly driven by pathogenic mutations in sarcomeric proteins (5). The remarkable case described by Pham et al. involving a mother and daughter, both harboring the same mutation in the cardiac Troponin T (TNNT2) gene, demonstrates the incomplete penetrance and variable expression within TNNT2-positive HCM families. Despite their shared genetic background, the strikingly different disease presentations in these individuals underscore the challenges in predicting clinical outcomes based solely on genotype. In a separate case, Li et al. described a 14-year-old girl with HCM and sudden cardiac arrest, whose whole-exome sequencing demonstrated a novel heterozygous ALPK3 gene variant with severe clinical implications. This emphasizes the pivotal role of genetic testing in diagnosing HCM and guiding familial screening protocols. The report emphasizes the importance of closely monitoring, assessing risks, and evaluating genetic background to effectively manage HCM, particularly in identifying modifiable risk factors and necessitating timely interventions such as implantable cardioverter defibrillator (ICD) placement. Furthermore, Hamidi et al. presented an intriguing case of an 18-year-old woman with non-obstructive HCM who experienced sudden cardiac arrest due to ventricular fibrillation (VF) and subsequent sinoatrial node (SAN) arrest during VF episodes. Identifying a MYH7 gene variant as causative underscores the rarity of SAN arrest in HCM cases and highlights the crucial role of comprehensive electrophysiological assessment in managing complex arrhythmic presentations in HCM patients. This case serves as a reminder of the need for appropriate ICD selection in such scenarios. In summary, these case reports emphasized the importance of recognizing the variability in disease expression and the need for comprehensive risk assessment and management approaches to improve outcomes in HCM patients.

Myocarditis is a significant contributor to non-ischemic cardiomyopathy, ranging from acute heart failure to cardiogenic shock and ventricular arrhythmias (6). The unpredictable nature of this condition underscores the importance of early detection and appropriate management to improve patient outcomes. In a study by Brown et al., three cases of viral myocarditis suspected to be caused by coxsackievirus B were highlighted. Despite the initial concern for cardiogenic shock, all three patients showed positive outcomes with significant clinical improvement and restoration of left ventricular ejection fraction. This underscores the potential for a favorable short-term prognosis in cases of coxsackie myocarditis with appropriate treatment and close monitoring. Zhu et al. presented a case of a young man who faced fulminant myocarditis and cardiogenic shock due to M. pneumoniae infection. The case emphasizes the importance of atypical pathogens consideration, like M. pneumoniae, in patients with cardiac complications, especially in younger individuals. The successful utilization of intra-aortic balloon pump and veno-arterial extracorporeal membrane oxygenation in this case highlights the critical role of advanced cardiac support measures in managing severe manifestations of myocarditis. Cheng et al. reported a female with 53-year-old who initially misdiagnosed with fulminant myocarditis but later identified as pheochromocytoma. The successful treatment through alpha-blockade therapy and laparoscopic adrenalectomy showcased the importance of tailored management approaches in achieving significant clinical improvement. These cases illustrated the diverse presentations and challenges in diagnosing and managing myocarditis-related complications. They underscored the importance of considering various etiological factors, utilizing advanced cardiac support strategies, and maintaining clinical vigilance to optimize outcomes in patients with myocarditis-related cardiac issues.

The emergence of the coronavirus disease (COVID-19) caused by severe acute respiratory syndrome coronavirus 2 (SARS-CoV-2) has sparked a worldwide pandemic (7–9). Among the extrapulmonary manifestations of COVID-19, two intriguing cases shed light on the diverse spectrum of unexpected cardiovascular abnormalities linked to SARS-CoV-2 infection, emphasizing the critical importance of vigilant monitoring and comprehensive understanding of such complications. Yamamoto et al. unraveled a unique instance of COVID-19-related acute pericarditis evolving into subacute hemorrhagic pericardial tamponade, initially masquerading as malignancy-induced cardiac distress. The intricate diagnostic journey underscores the challenging nature of identifying pericardial involvement in post-COVID-19 patients and the significance of exploring varied cytomorphological and histological features to reach a precise diagnosis. Zhang et al. delved into the rare occurrence of arterial pseudoaneurysms associated with SARS-CoV-2 infection, shedding light on the potential complexities of vascular abnormalities in the COVID-19 context. This study presented a patient developing pulmonary and gallbladder artery pseudoaneurysms after SARS-CoV-2 infection, serving as a crucial step in understanding the enigmatic pathophysiological mechanisms underlying SARS-CoV-2-associated artery pseudoaneurysms. Early diagnosis and prompt surgical intervention can greatly improve the long-term outlook for patients.

Two highlighted case reports underscored the complexity of Kounis syndrome (KS), characterized by the confluence of an allergic or hypersensitivity reaction and acute coronary syndrome. A clear consensus on diagnosing and treating KS remains elusive, with challenges in effectively identifying and managing KS patients in a timely manner. Sun et al. demonstrated the diagnostic challenge in a patient without coronary artery disease or allergies-related disease history, emphasizing the importance of recognizing KS in such scenarios. A Type II KS diagnosis was confirmed by a significant allergic reaction and widespread peripheral vasodilation during angiography. Tang et al. further illustrated the life-threatening potential of KS, where a patient developed severe symptoms after receiving intravenous flurbiprofen. The lack of significant stenosis or thrombus on coronary angiography, leading to a diagnosis of Type I KS, highlights the heterogeneity of this syndrome. These cases underscored the need for heightened clinical awareness and a multidisciplinary approach in managing KS, as timely recognition and appropriate treatment can be crucial for patient outcomes.

The collection of case reports presented in this research topic offers invaluable insights into the diagnosis and management of several rare and complex cardiovascular conditions, further expanding our clinical knowledge and improving patient care. Wang et al. highlighted the diagnostic challenge of primary pericardial mesothelioma (PPM), a rare malignant cancer with a poor prognosis. The case underscored the importance of a multidisciplinary approach, with consideration of the patient's occupational asbestos exposure history and a prompt biopsy leading to an early diagnosis. The successful use of a bevacizumab-based chemotherapy regimen as the first-line treatment is a promising development. Hali et al. emphasized the importance of recognizing coronary artery mycotic aneurysms (CAMA) in the context of infective endocarditis. While mycotic aneurysms are well-known in the cerebrovascular system, CAMA remains a rare but critical complication that deserves greater clinical attention. The case described a 42-year-old man with diabetes, cerebellar infarction and endocarditis caused by Viridans Streptococci. Diagnostic imaging revealed aneurysmal dilatation of the left main coronary artery, confirming CAMA. Due to high surgical risk, the patient was managed conservatively with prolonged antibiotic therapy, leading to a favorable 1-year outcome. Hattori et al. shed light on a rare and perplexing condition of cardiocerebral infarction (CCI), where simultaneous cerebral and coronary artery embolism occur, often with a fatal outcome. The autopsy findings in this 92-year-old patient revealed a critical role of atrial fibrillation-induced left atrial appendage thrombi as the common etiology for the cerebral and coronary artery embolism. Another pathological study was warranted to establish pathological mechanisms and Intervention strategies of CCI. Tinzin et al. reported the importance of recognizing the risk of delayed cardiac rupture following thoracic trauma, even in patients who seem to be recovering well initially. This rare complication can lead to sudden death, emphasizing the need for heightened vigilance and appropriate management strategies in such cases. Valencia et al. described a successful use of venoarterial extracorporeal membrane oxygenation (VA-ECMO) as a rescue for refractory vasoplegic shock following an accidental treprostinil overdose in a patient with pulmonary arterial hypertension. ECMO helps maintain organ perfusion and allows for rapid recovery similar to drug elimination. It's important to continuously monitor the patient to determine the best time to remove them from ECMO to avoid complications. Gao et al. reported an elderly patient with Sheehan's syndrome, who developed severe hyponatremia after a percutaneous coronary intervention, requiring prompt hydrocortisone administration to rapidly alleviate symptoms. This highlights the critical importance of stress-dose glucocorticoid supplementation before such procedures, especially in patients with adrenal insufficiency. Comprehensive endocrinological management is crucial for this patient population.

In summary, case reports in this special issue provided invaluable insights into the diagnosis and management of rare and complex cardiovascular conditions, emphasizing the importance of a multidisciplinary approach and evidence-based interventions to improve patient outcomes in these challenging scenarios.
